# Are early career family physicians prepared for practice in Canada? A qualitative study

**DOI:** 10.1186/s12909-023-04250-z

**Published:** 2023-05-24

**Authors:** Monica Aggarwal, Reham Abdelhalim

**Affiliations:** 1 Dalla Lana School of Public Health, University of Toronto, Toronto, ON Canada; 2 Institute of Health Policy, Management and Evaluation, University of Toronto, Toronto, ON Canada

**Keywords:** Preparedness for practice, Family medicine, Family physicians, Postgraduate training, Medical education

## Abstract

**Background:**

In Canada, the College of Family Physicians of Canada (CFPC) introduced Competency Based Medical Education to prepare and train family medicine residents to be competent to enter and adapt to the independent practice of comprehensive family medicine. Despite its implementation, the scope of practice is narrowing. This study aims to understand the degree to which early career Family Physicians (FPs) are prepared for independent practice.

**Method:**

A qualitative design was used for this study. A survey and focus groups were conducted with early-career FPs who completed residency training in Canada. The survey and focus groups examined the degree of preparedness of early career FPs in relation to 37 core professional activities identified by the CFPC’s Residency Training Profile. Descriptive statistics and qualitative content analysis were conducted.

**Results:**

Seventy-five participants from across Canada participated in the survey, and 59 participated in the focus groups. Early career FPs reported being well prepared to provide continuous and coordinated care for patients with common presentations and deliver various services to different populations. FPs were also well prepared to manage the electronic medical record, participate in team-based care, provide regular and after-hours coverage, and assume leadership and teaching roles. However, FPs reported being less prepared for virtual care, business management, providing culturally safe care, delivering specific services in emergency care hospitals, obstetrics, self-care, engaging with the local communities, and conducting research activities.

**Conclusions:**

Early career FPs do not feel fully prepared for practice in all 37 core activities in the Residency Training Profile. As part of the introduction of the three-year program by the CFPC, the postgraduate family medicine training should consider providing more exposure to learning opportunities and developing curricula in the areas where FPs are unprepared for practice. These changes could facilitate the production of a FP workforce better prepared to manage the dynamic and complex challenges and dilemmas faced in independent practice.

**Supplementary Information:**

The online version contains supplementary material available at 10.1186/s12909-023-04250-z.

## Background

Family physicians (FPs) are integral to the Canadian healthcare system since they are the first point of contact with the system and are expected to provide a comprehensive scope of services to patients [1, 2]. In 2010, the College of Family Physicians of Canada (CFPC), the accrediting and certifying body for family medicine in Canada, adopted the Competency-Based Medical Education with the Triple C-Competency Based Curriculum [3]. The objective of the curriculum was to prepare and train family medicine (FM) residents to be competent to enter and adapt to the independent practice of comprehensive family medicine anywhere in Canada [4]. The Triple C curriculum is designed to foster an environment enabling learners to acquire FM-specific competencies through learning experiences provided in FM contexts [5]. The curriculum promotes training environments that are **C**omprehensive, allow for **C**ontinuity, and are **C**entered in FM [4] The transition from time-based to competency-based education was intended to teach and assess the specific competencies needed for practice [6], with the achievement of key milestones during and at the end of residency training [5]. The 2-year length of training remained the same despite demographic changes, patient and societal expectations, medical advances, and new technologies, which have contributed to the demanding and complex role of FPs.

However, despite the implementation of the Triple C Curriculum, comprehensive care is declining in Canada [7–10]. Many FM graduates do not (or do not intend to) provide comprehensive care [11–13]. Service volume [14–17], working full-time [12], patient panel sizes [12] are decreasing, and fewer FPs are providing services at non-office-based locations [10, 18]. The declining trend in the scope of practice has raised important questions about the degree to which the Triple C Curriculum prepares FPs for the independent practice of comprehensive family medicine [10, 19]. Insufficient preparedness for practice is related to stress and burnout [20, 21], which may limit comprehensive practice [8]. Other factors that may impact decisions about comprehensive practice include personal, workplace, environmental, and population factors [22].

In 2018, the CFPC published the Family Medicine Professional Profile (FMPP) to clarify and describe the collective vision of FPs providing comprehensive care close to home. The eight professional responsibilities identified in the FMPP include primary care, maternal and newborn care, home and long-term care, emergency care, hospital care, advocacy, leadership, and scholarship [23]. In 2021, the CFPC released the Residency Training Profile (RTP) to define the broad scope of training required to prepare FPs to provide comprehensive care and meet the evolving health care needs of patients and communities in Canada [24]. The RTP is based on the FMPP and details the core professional activities (CPAs) or tasks that FM residents are expected to learn by the end of training. This study aimed to provide insights into the perspective of early career FPs concerning their preparedness for practice after completing the Triple C Curriculum in Canada. This study examines the degree to which early career FPs are prepared for the independent practice of comprehensive family medicine as defined by the FMPP and RTP.

## Methods

### Study design

This paper reports on a subset of the data collected as part of the larger multi-method research project that studied the preparedness of early career FPs. For this paper, we utilized a qualitative descriptive design to examine the study question [25, 26]. The study took place in two stages. The first stage consisted of an online survey. The objective of the survey was to identify the characteristics of the participants and determine the degree to which FPs were prepared for the domains in the FMPP and the CPAs for primary care outlined in earlier drafts of the CFPC’s RTP [24]. This information helped to contextualize and inform the direction of the second phase. The second phase of the study involved conducting focus groups. The purpose of the focus groups was to gain an in-depth understanding of the FPs’ degree of perceived preparedness to practice, the barriers and facilitators of preparedness, and identify educational strategies to improve the FM training program in Canada.

### Study participants

All participants were English, or French-speaking certified early career FPs who had completed residency training in Canada and had been practicing FM for 2–5 years since graduation. Participants were recruited from all Canadian jurisdictions. To be eligible for this study, participants had to be: (1) certified early-career FPs; (2) completed residency training in Canada; and (3) practicing for 2–5 years since graduation. Exclusion criteria included FPs who did not complete residency training in Canada and were less than two years or over five years into practice. Recruitment approaches included: snowball sampling techniques [27, 28], posting study material on social media websites (e.g., Facebook, CFPC website), broadcasting to various CFPC committees and working groups, requesting key informants to share recruitment letters with eligible participants, inviting the CFPC’s First Five Years in Practice (FFYP) Committee to participate and nominate early-career FPs. Eligible members of the CFPC’s First Five Years in Practice (FFYP) Committee, including the Chair, were the first group to be invited to participate in the focus groups. In addition to the invitation to participate, we asked the Chair and each member to nominate three more early-career FPs that fit the inclusion/exclusion criteria, plus one early-career FP practising in the Northwest Territories, Yukon, or Iqaluit. Permission to share contact information was obtained by the nominees before the name or contact information was provided to the research team. The eligibility of participants was screened by the research assistant [RA], who contacted potential participants between September 2020 and May 2021 via email with a letter of invitation and consent form in both official languages (English and French). Study participants that agreed to be contacted by the research team were asked to nominate three more FPs based on the inclusion/exclusion criteria, regardless of their own participation decision. Participants that agreed to participate in the study were asked to complete a survey. The collection of this information assisted with purposeful sampling and ensuring diversity in the composition of the focus group. All participating FPs completed the survey and were invited to participate in the focus groups regardless of survey responses.

We used purposive sampling with a maximum variability technique to ensure participants’ representativeness to the broader population of early-career FPs [29]. This variability was sought in participants’ age, sex, years in training, university of training, practice location, type of services provided in practice, involvement in different practice models, interprofessional teams, and academic activities. The snowballing technique was continued until we achieved a heterogeneous group of 6–8 participants per focus group with an appropriate mix of participants reflecting the diverse perspectives across all focus groups. In total, invitations were sent to 160 early-career FPs. Only those who provided consent participated in the study.

### Data collection

In the first phase, a 20-minute online survey was sent to early career FPs using REDCap (Refer to **Supplementary Material 1**). This survey aimed to collect basic demographic information that helped the research team apply the maximum variability technique to the focus groups. The survey additionally gathered data on the degree of preparedness of early career FPs with 37 CPAs in the nine domains of the FMPP. These nine domains are: attend to practice, comprehensive and inclusive primary care, maternal & newborn care, emergency care, home & long-term care, hospital care, advocacy, leadership, and scholarship. The response option for the survey items was a 5-point Likert scale (extremely well prepared, well prepared, prepared, not very well prepared and unprepared). The survey was pre-tested with four early career FPs to ensure clarity and consistent interpretation of questions. No changes were made to the survey because of the pre-testing. The survey was available in English and French-based on the preference of participants. Participants were given two weeks to complete the survey. Several reminders were sent to enhance the response rate, as recommended by Dillman [30].

We conducted 12 virtual focus groups across Canada. Focus groups were organized by jurisdiction. One focus group took place in each of the six provinces (Alberta, British Columbia, Manitoba, Newfoundland and Labrador, Quebec, and Saskatchewan), and one combined focus group represented New Brunswick, Prince Edward Island and Nova Scotia (Maritimes). Due to scheduling issues, two focus groups were done for Ontario and Northern Canada (Territories). We also conducted two focus groups for Quebec (one in English and one in French) based on the language preferences of the participants.

The focus groups were conducted using a web-based enabled videoconference, the MS Teams platform. A semi-structured interview guide (Refer to **Supplementary Material 2**) and the FMPP were provided to participants before the focus groups. The interview questions explored participants’ perceptions concerning the definition of preparedness to practice, areas of preparedness for practice, educational factors that shaped preparedness for practice, and their intentions and practice choices. The survey data results guided focus group discussions about the areas of most or least preparedness in each jurisdiction.

Each focus group was scheduled for a specific time and date convenient for participants. Focus groups lasted anywhere between 90 and 110 min. Participants were also allowed to include their thoughts and feedback in the chat box, which was reviewed and analyzed. Two experienced qualitative researchers [MA and RA] conducted the focus groups. One Quebec focus group was conducted in French and was facilitated by a French-speaking facilitator. Notes were taken during the focus groups, and both interviewers met after each focus group to debrief and discuss themes. The notes helped the authors determine when enough data had been collected to reach data saturation and suggested no further focus groups were needed to yield new findings [31].

The virtual focus groups were recorded, transcribed, and securely deleted once coding was completed. Participants were offered a $50 gift card honorarium to appreciate their time. The study was approved by the University of Toronto’s Health Research Ethics Board (protocol #39,077).

#### Analysis

Survey data analysis involved descriptive statistics for participants’ characteristics and assessment of perceived preparedness with the FMPP. To examine early-career FPs’ perceived preparedness, we used Likert charting to describe the percentages of those who were prepared versus unprepared for each professional activity.

Focus group data were analyzed using qualitative content analysis, a form of analysis that summarizes the informational contents of the data [26]. Focus group analyses were guided using a previously constructed codebook informed by the FMPP as the guiding framework. New codes were added iteratively as they arose and grouped to form categories. The content analysis followed the four-step process described by Krippendorff [32]: [1] the sampling of data from a medium or a group of participants; [2] unitizing data in terms of words or propositions, such as using excerpts, examples, or quotes; [3] contextualizing data by exploiting the researcher’s comprehension of the context from which the data have been derived; and [4] relating the findings to a research question [32].

NVivo 12 software was used to facilitate coding and help analyze the data [33]. Two authors [MA and RA] coded transcripts and met several times to resolve discrepancies until consensus was attained. In the focus groups where participants shared their views and thoughts via the chat box, the comments in the chat box were analyzed using the same methodology for analyzing the transcripts. Data were analyzed by two authors [MA and RA], who met several times to agree on the study’s findings.

The researcher is often the data collector and data analyst within qualitative research, allowing researcher bias [34]. We involved participants in checking and confirming the results [35]. A summary of the findings was shared with four early-career FPs. Participants were asked to provide feedback on whether the results aligned with their experiences. Two participants provided feedback and indicated that they agreed with the findings. Two participants did not respond to our request for feedback.

## Results

To examine the degree of preparedness of early-career FPs regarding the CPAs concerning the FMPP, we analyzed and integrated the survey results with the findings from the focus groups. Seventy-five participants completed the survey, and 59 participated in the focus groups. Sixteen survey respondents could not participate in the focus groups due to pandemic response or personal responsibilities. The characteristics of the 59 participants are described in Table 1**- Characteristics of Participants**.

**Table 1:** Characteristics of participants that completed the survey

	*AB*	*BC*	*MB*	*Maritimes*	*NL*	*ON*	*QC*	*SK*	*Territories*	*Total*
Number of Participants	10	9	7	6	8	13	8	7	7	75
Age (mean)	33.2	34.7	33.4	37.8	33.8	34.2	32.1	33.1	35.0	34.1
Sex										
*Female*	6	8	3	6	7	6	6	4	6	52
*Male*	4	1	3		1	7	2	3	1	22
*Prefer not to answer*			1							1
Years in Practice										
*Two*		2	1		3		1			7
*Three*	10	6	4	4	2	10	4	1	4	45
*Four*		1	2	1	1	3	3	3	1	15
*Five*				1	2			2	2	7
*Missing*								1		1
Place of Residency Training										
*Trained in Canada*	12	7	7	4	11	19	7	7		74
*Trained partially outside Canada*							1			1
Practice Location										
*Inner City*		1	1	1		1				4
*Urban/ Suburban*	4	6	2	2	2	4	4	3	1	28
*Small Town*	2			2		1	2	1		8
*Rural*	2		1		4	3		1		11
*Remote/ Isolated*						1			2	3
*Mixture of environments*	2	2	3	1	2	3	2	2	4	21
Practice Model										
*Solo practice*				1	2					3
*Group physician practice*	7	6			4	4	5	2	3	31
*Interprofessional team-based practice*		2	3	4	2	7	2	4	3	27
*Mixed practice*	1	1	3	1						6
*Other*	2		1			2	1	1	1	8
Practice Type										
*Comprehensive in one setting*	1	2		1		4	4			12
*Comprehensive in multiple settings*	7	3	5	3	4	6	1	3	5	37
*Comprehensive with a special interest*		4			1	1	2	1	1	10
*Focused*	1		1	1		1	1	1	1	7
*Other*	1		1	1	3	1		2		9
Academic affiliation										
*Yes*	8	6	6	4	5	9	7	7	6	58
*No*	2	3	1	2	3	4	1		1	17

The results of the level of preparedness for the 37 CPAs in the nine domains are presented in Table 2**- level of preparedness for the 37 CPAs in the nine domains.** Below, we report our findings from the focus group regarding their self-reported preparedness and unpreparedness.

**Table 2:** Level of preparedness for the 37 CPAs in the nine domains.

Core Professional Activity and Domains	Level of Preparedness (number (%) of respondents) defined as prepared (i.e., prepared, well prepared or extremely well prepared) or unprepared ( i.e., not very well prepared or unprepared).		
**Attend to Practice**	Prepared	Unprepared	Unreported
Provide access to care by maintaining a regular scheduled after-hours coverage as part of an overall system of care to the practice	61 (81)	13 (17)	1 (2)
Provide virtual care as part of a system of access and continuity for the practice	12 (16)	62 (83)	1 (1)
Manage the ‘total care’ of patients providing continuity, follow-up and coordination	73 (97)	1 (1.5)	1 (1.5)
Assess and plan for the care needs of the practice in the context of the local community	56 (75)	18 (24)	1 (1)
Maintain an electronic medical record for each patient as part of a system of medical documentation for the practice	56 (75)	18 (24)	1 (1)
Attend to practice and personal business functions	32 (42)	42 (56)	1 (1)
Support and engage with patient safety processes	58 (77)	15 (20)	2 (3)
Participant in collaborative and team-based care	70 (93)	4 (5)	1 (1)
Manage self-care to support personal well-being and sustainable practice	46 (61) =	28 (37)	1 (1)
**Comprehensive and inclusive primary care**			
Provide reproductive care	65 (87)	7 (9)	3 (4)
Provide comprehensive continuity-based primary care for children and youth	68 (91)	7 (9)	0 (0)
Provide comprehensive continuity-based primary care for adults	75 (100)	0 (0)	0 (0)
Provide comprehensive continuity-based primary care for the elderly	68 (91)	7 (9)	0 (0)
Provide primary palliative and end-of-life care	62 (83)	13 (17)	0 (0)
Manage patients with complex and co-morbid illnesses	62 (83)	7 (17)	0 (0)
Provide primary care that addresses the health care needs of diverse peoples as part of a commitment to health equity	64 (85)	11 (15)	0 (0)
Provide culturally safe primary care that addresses the specific health care needs of First Nations, Inuit and Metis people	44 (59)	3 (41)	0 (0)
Perform common minor/office procedures (see Core Procedures list)	65 (87)	10 (13)	0 (0)
**Emergency care**			
Provide antepartum care	62 (83)	7 (17)	0 (0)
Manage a low-risk labour and delivery	48(64)	27 (26)	0 (0)
Provide postpartum care	67 (89)	8 (11)	0 (0)
Provide newborn care in the hospital and community	66 (88)	9 (12)	0 (0)
Perform common intrapartum care procedures (see Core Procedures list)	47 (63)	28 (27)	0 (0)
Assess and manage patients of all ages with common urgent and emergent presentations in all settings	65 (87)	9 (12)	1 (1)
Assess and stabilize patients of all ages with life-threatening, high-acuity presentations in all settings	47 (63)	28 (27)	0 (0)
Perform commonly required emergency procedures (see Core Procedures list)	46 (61)	29 (39)	0 (0)
***Hospital care***			
Provide medical care in the hospital as the ‘Most Responsible Physician’	66 (88)	9 (12)	0 (0)
Provide surgical assistance in the operating room	48 (64)	27 (36)	0 (0)
Perform common in-hospital procedures (see Core Procedures list)	50 (67)	23 (31)	2 (2)
**Advocacy**			
Work with patients to assess and address their social determinants of health	61 (81)	14 (19)	0 (0)
Engage with the local community to understand and improve health conditions and access to care	47 (63)	28 (27)	0 (0)
L**eadership**			
Provide leadership in everyday professional practice	59 (79)	15 (20)	1 (1)
**Scholarship**			
Maintain and enhance knowledge to provide care that is evidence-informed and responds to practice needs	72 (96)	3 (4)	0 (0)
Participate in QI activities as part of practice improvement	58 (77)	17 (23)	0 (0)
Participate in research activities as part of practice improvement	50 (67)	25 (23)	0 (0)
Teach and supervise learners in everyday practice functioning as a ‘clinical coach’ (per FTA Framework)	61 (81)	14 (19)	0 (0)
**Home & Long-Term Care**			
Provide primary care for patients with unique and complex medical needs in the home, long-term care facility and other community-based settings	57 (76)	17 (23)	1 (1)

### Attend to practice

We found that participants reported being prepared for maintaining an electronic medical record for each patient, team-based care, managing the continuous and coordinated care of patients, providing access to care through regular and after-hours coverage.

Participants were least prepared for providing virtual care. These findings were reflected in the focus groups, where participants discussed their lack of preparedness for virtual care, especially during the COVID-19 pandemic. A participant said:

*“So back in residency, It wasn’t during the pandemic, so we didn’t really have any telemedicine, virtual care. Everything’s in person. And then now everything is all virtual. And I think something that I wasn’t prepared for is I get a lot of people who might seek opioids, benzos, and they’re asking for prescriptions of those all over the phone. And you can’t really see them. You can’t assess them. […] And so just having everything be online now, it’s just harder to do physical exams, and it’s harder to screen people if they’re coming in for opioid-seeking purposes.”* (Ontario 2nd FG)

Many of participants reported being unprepared for business functions. The lack of preparedness for business management, financial management, and administration emerged as the most prominent theme in the focus groups. Participants discussed their lack of preparedness on the processes involved in owning and managing a business or practice:

*“Like I just found that residency did not at all prepare me for starting my own practice of my own. Like no part of it. Like from the billing to…like any of it, really. Because what I was exposed to in residency, even though I trained where I set up my practice, it’s still like, you know, as a resident, you’re often sheltered from that aspect, right.”* (Newfoundland FG)

Other participants talked about their lack of preparedness for dealing with time-consuming administrative processes such as insurance forms:

*“Insurance forms or disability paperwork for a patient. I really wish that even if there was one god-awful lecture on it where I could have taken some tips forward or something would have stuck in my brain, maybe there would have been something that better prepared for the vast majority of things that we get asked to fill out on a daily basis.”* (Alberta FG)

Many participants also reported feeling unprepared to manage self-care to support well-being and sustainable change.

### Comprehensive and inclusive primary care

In the comprehensive and inclusive primary care domain, many participants felt prepared for providing various services (common minor/office procedures, reproductive care, palliative care), comprehensive care to various populations (elderly, children, adults, diverse and medically complex populations). Participants were less prepared to provide culturally safe care that addresses the specific health care needs of First Nations, Inuit, and Metis people.

One focus group participant shared:

“*I noticed in residency how not only how ill-prepared you were to deal with issues of social determinants of health or providing culturally safe care but it’s more how disempowering it is in this situation. Like I knew I had lots to learn starting to practice in a First Nation remote community.*” (Quebec 2nd FG)

### Emergency care

Fourty six percent of participants reported being prepared for managing patients of all ages with common urgent and emergent presentations in all settings. However, participants said they were unprepared to perform commonly required emergency procedures and assess and stabilize patients of all ages with a life-threatening, high-acuity presentation in all settings.

A participant discussed their challenges:

*“I didn’t realize that I wasn’t ready for emergency care. That was actually what I planned on my entire practice being until I tried it. I did electives and extra rotations in ICU and emergency. And then as soon as the support is taken away and you’re on your own, it feels very different. And I didn’t feel comfortable managing that.”* (Alberta FG)

### Hospital care

In the hospital care domain, many of the participants reported being prepared to be the “most responsible physician.” However, respondents reported being unprepared to perform common in-hospital procedures, and to provide surgical assistance in the operating rooms.

Participants shared their stories on the lack of preparedness in performing surgical procedures and handling life-threatening situations in the hospital:

*“I felt very uncomfortable with the thought of intubating anybody or participating in, let’s say, a resuscitation from a gunshot wound because the hospital I trained at just didn’t get patients like that. It wasn’t a trauma centre. And those people were usually diverted elsewhere.”* (Territories 1st FG)

### Advocacy

In the advocacy domain, participants reported feeling prepared to work with patients to assess and address their social determinants of health. In comparison, participants reported being unprepared to engage with the local community to understand and improve health conditions and access to care.

A participant discussed her lack of awareness of the need to advocate in practice:

“*But the other thing that I wasn’t picturing as part of my process, and it’s such a big part of my life, is the advocacy work that has really come in an unavoidable way, it seems, over the past few years. Both advocating for patients but mostly advocating for policy change at a local and a provincial level.”* (Manitoba FG).

### Leadership

In the leadership domain, many participants reported being prepared.

### Scholarship

In the scholarship domain, respondents felt prepared to teach and supervise learners. More than half of the participants reported being prepared to maintain and enhance knowledge to provide evidence-informed care and respond to practice needs. Participants were unprepared to participate in research activities for practice improvement and participation in quality improvement activities.

### Home & long-term care

Many participants reported feeling prepared to provide primary care in-home and long-term facilities, while some reported feeling unprepared.

A focus group participant discussed their experience:

*“I think home care or long-term care was another one that was very hit and miss. And part of it is just because I think like the staff that I worked with during my residency, they did home care or long-term care, but it was one of those things that was kind of off the side. And so, they had so many other priorities.”* (Saskatchewan FG)

### Maternal and newborn care

With maternal and newborn care, participants reported feeling prepared for providing antepartum, postpartum and newborn care. However, participants reported they were unprepared to manage low-risk labour and delivery and perform common intrapartum care procedures.

The lack of preparedness for this domain also emerged as a dominant theme in the focus groups:

*“And a vacuum was one thing I’d only applied once under the supervision of a supervisor in my OB residency. So, when I was faced with a situation where I had put on a vacuum independently, I felt unprepared, having only done it once before.”* (Alberta FG)

### Non-clinical areas

Participants also reported being unprepared to manage difficult patients, having multiple responsibilities and multiple concerns from patients and dealing with failure. Some participants discussed their experience with difficult patients and their families and complex cases:

*“What I was least prepared for was when I…or my clinic started receiving letters in which the daughter of one of my older patients was criticizing me. And it was the sort of situation where I couldn’t really respond or talk to her about it because, you know, the patient was competent and didn’t want the daughter involved. And I thought I was doing the best I could..” (Ontario 2nd FG)*

*“I think that a lot of times in residency, we were very much shielded from difficult requests from patients by saying, you know, it’s up to my preceptor, I’ll let my preceptor sort out your note or your form. And I struggled a lot to begin with, feeling unprepared with how to manage requests that I felt were inappropriate like forms or requests that I just didn’t think were medically indicated. […] For myself, I was feeling pretty unprepared trying to manage or trying to balance my boundaries and my values as a physician with what some patients were requesting of me.” (Alberta FG)*

A participant discussed how the program did not prepare graduates to address the multiple responsibilities that are involved in their role as an FP:

*“I think some of the things to add onto that would be like not so much time management[…]You’re the one who has to do everything. And then trying to like balance that with all the other things you want to do….So realize all the extra that we were protected from - the lawyers’ letters, the forms, the calls, when people would call and say, “Can I have this, can I have that?“ Just managing people who want your time and attention.” (*Newfoundland FG*).*

Another participant indicated how the training program set unrealistic expectations of what a family doctor can deliver regarding patient services. This is further reflected in their assessments and examinations:

*“I often felt on my end I was expected to address if I had a complicated patient with 10 problems, they came in with [..]full assessments of each problem. And it’s just it’s not realistic. It’s often in your own practice, you might deal with two, three issues, do a few questions for some other issues to make sure that it’s not urgent, urgent, and you’re not letting someone walk away with a very urgent issue. And then maybe bring them back a few weeks later and address those other issues. And I don’t think we were taught in residency how to comprehensively address some issues, triage, and safely address others without fully assessing others.”* (Quebec 2nd FG).

One participant felt unprepared to deal with failure, advocate for oneself and patients and to be adaptable and resilient:

*“I think another thing that residency could prepare us better for is things like proper ways of dealing with failure, because I still feel like there’s an attitude of, well, this is just kind of how it is and you have to suck it up, and everyone has to go through this. And like advocating I guess for system change and how to protect yourself within the system, protect your patients within the system. [..]think it surprises everybody regardless if you go into fee-for-service practice or if you go into salary-based practice, if you limit your scope or if it’s a wide scope, if you went urban, if you went rural, I think that some of those things that come up, I think it still surprises you. […]I guess, in hidden curriculum, if anything.” (Saskatchewan FG)*

## Discussion

The Triple C curriculum was implemented to produce FM graduates prepared to enter and adapt to comprehensive family medicine practice in any community in Canada. However, the scope of practice has narrowed in Canada [7, 10, 36, 37]. These trends are worrisome as many Canadians already do not have an FP [38–40]. This study explores the degree to which early career FPs are prepared for independent practice.

This study found that early career FPs were well prepared to manage and provide continuous and coordinated care for patients with common presentations and deliver various services (office procedures, reproductive care, end-of-life care) to different populations (children and youth, adults, elderly, complex patients with multiple morbidities). FPs were also well prepared to maintain an electronic medical record, involvement in team-based care, provide regular and after-hours coverage, and assume leadership and teaching roles. These findings may reflect that FPs received more exposure to these domains during FM training.

However, FPs were less prepared for virtual care, business management, providing culturally safe care (i.e., First Nations, Inuit, and Metis people), delivering specific services in emergency care hospitals, obstetrics, self-care, engaging with the local communities, and conducting research activities. Previous studies have also shown a lack of preparedness for business management, the development of procedural skills for common emergency presentations, and frustrations with engaging in health advocacy during training in Canada [9, 41–44]. FPs also reported that they were not prepared to manage conflict with patients and their families, address the needs of complex patients, handle multiple responsibilities, and psychologically address failure. The lack of preparedness for virtual care, business management, self-care is not surprising since there is less focus on these areas in the curriculum [45, 46]. Furthermore, the exposure to emergency departments, hospitals and obstetrics, community engagement, research and diverse populations may be limited in a two-year program [47, 48].

Our findings indicate there are opportunities to improve the FM curriculum in Canada, especially as the CFPC embarks on the implementation of the three-year program. Previous studies indicate that increasing exposure to clinical and non-clinical domains during training increases preparedness for practice [48–50]. To build the competencies and capabilities for adaptability and resilience, FM programs program could provide more exposure to various primary care settings in different geographical locations, populations, and environments and opportunities, including virtual care. This would provide more opportunities to deliver continuous care, address multiple concerns and deal with difficult patients. Exposure to extensive rural placements in training has been associated with a broader scope of practice and practice in rural practice locations [51–53]. Exposure to rural practices where resources are less available can build resiliency and the ability to deal with failure [54].

FM programs should also include opportunities for flexible and customary placement time in obstetrics, emergency departments, and hospital care to acquire greater proficiency and confidence. Academic half-days can improve resident medical knowledge acquisition and increases learner satisfaction [55]. Formal lectures, didactic sessions, rotations in primary care settings, and simulation-based learning have been used for business management [56]. Training programs should include formal curriculum to develop learning skills in business or practice management, virtual care and health care policy as well as to balance the competing professionalism demands of altruism and self-care [57,58, 59]. Stimulation based learning would be particularly helpful in providing exposure to complex situations and increasing confidence in rare situations [60]. In addition, culturally competent education can be implemented for equity-deserving populations [61].

Preceptors and mentorship have a key role in influencing preparedness for practice. Previous studies have shown that the attitudes and behaviours of clinical preceptors towards patients and learners influenced the behaviours of trainees [62]. Early career FPs indicated that their preparedness for practice could have been improved by more concerted efforts to have preceptors provide longitudinal exposure to patients from various populations and work settings to help develop their knowledge, skills, competencies and capabilities. In addition, FPs suggested a formalized mentorship program that matches residents with mentors during and after the completion of the program to facilitate the transition to practice. Thus, any changes to the three-year program should be accompanied by providing sufficient support for the FM program, faculty, and mentors. In addition, programs should be standardized so that residents receive similar training opportunities and include requirements for social accountability through continuous quality improvement [63].

### Limitations

As with all research, our findings should be considered within the context of their limitations. Respondents’ reflections on their experiences and the expectations they faced in earlier years may have caused recall bias. Similarly, our findings are limited to early-career FPs who agreed to participate in the study and included a small sample for the survey. Our study is not representative of the broader population of FPs in Canada. For example, our study did not include non-binary or other gender-diverse physicians, nor did we have older participants. We tried to mitigate this limitation by including a diverse sample that showed variability in age, sex, practice type, model, and location to provide rich results. The spectrum of participants from various regions provides grounds for the transferability of findings. Given the anonymity of the survey, we could not attribute the survey responses to focus group participants. Canada was facing a sharp rise in COVID-19 cases while conducting member checking, which affected their ability to provide feedback. Our findings represent a single point in time and lack the longitudinal perspective to reflect variability in experiences over time.

## Conclusions

Early career FPs did not feel fully prepared for all 37 core activities outlined by the CFPC’s FMPP and RTP, including business management, providing virtual care, culturally safe care, care in emergency departments, hospitals, and obstetrics and research. Providing trainees with more exposure to learning in specific areas and settings and introducing a new curriculum may facilitate the production of an FP workforce better prepared for practice. However, this must be accompanied by supporting FM programs, preceptors, and mentorship programs. Together, these changes could facilitate the production of a FP workforce better prepared for managing the dynamic and complex challenges and dilemmas faced in independent practice.

## Electronic supplementary material

Below is the link to the electronic supplementary material.

Supplementary Material 1

Supplementary Material 2

## Data Availability

The manuscript includes the data and study material.

## Abbreviations

CFPC: College of Family Physicians of Canada
FPs: Family Physicians
FMPP: Family Medicine Professional Profile
FM: Family Medicine
RTP: Residency Training Profile
