# Supplementary material for: Are early career family physicians prepared for practice in Canada? A qualitative study
Source: BMC Med Educ. 2023 May 24;23:370. doi: 10.1186/s12909-023-04250-z (PMC10206365; doi:10.1186/s12909-023-04250-z)
Supplement: Supplementary file 1 — Supplementary Material 1 [file 12909_2023_4250_MOESM1_ESM.docx]

**Supplementary Material 1: Survey**

1) Please enter the year you were born. ________________________

2) What is your gender?

( ) Female

( ) Male

( ) Non-binary

( ) Prefer not to answer

3) How many years have you practised family medicine in Canada, either full or part-time (minimum 20 hours per week) since completing your FM residency training?

( ) 3 years

( ) 4 years

( ) 5 years

4) At what university did you complete your FM RESIDENCY training?
( ) University of British Columbia

( ) University of Calgary

( ) University of Alberta

( ) University of Saskatchewan

( ) University of Manitoba

( ) University of Western Ontario

( ) McMaster University

( ) University of Toronto

( ) Northern Ontario School of Medicine (NOSM)

( ) University of Ottawa

( ) Queen’s University

( ) Université de Sherbrooke

( ) Université de Montréal

( ) McGill University

( ) Université Laval

( ) Dalhousie University

( ) Memorial University

( ) Outside Canada

5) Where is your practice located?

( ) British Columbia

( ) Alberta

( ) Saskatchewan

( ) Manitoba

( ) Ontario

( ) Quebec

( ) Nova Scotia

( ) New Brunswick

( ) Prince Edward Island

( ) Newfoundland and Labrador

( ) Yukon

( ) Northwest Territories

( ) Nunavut

6) Select the ONE statement that best describes the environment in which you are currently practicing family medicine?

( ) Exclusively/ predominantly inner city

( ) Exclusively/ predominantly urban/ suburban

( ) Exclusively/ predominantly small town

( ) Exclusively/ predominantly rural

( ) Exclusively/ predominantly remote/ isolated

( ) Mixture of environments (Please describe):

**Important Terms**

***For the purposes of the survey, comprehensive care describes the type of care family physicians provide (either on their own or with a team) to a defined population of patients across the life-cycle in multiple clinical settings (e.g., Office-based, hospital, in- home…) addressing a spectrum of clinical issues (from prevention to acute to chronic disease and palliative care).**

**Family Physicians with special interests: family doctors with traditional comprehensive continuing care family practices who act as the personal physicians for their patients and whose practices include one or more areas of special interest as integrated parts of the broad scope of services they provide; and**

**Family Physicians with focused practices: family doctors with a commitment to one or more specific clinical areas as major part-time or full-time components of their practices.**

7) Which of the following best describes the organizational model(s) you currently practice in?

(Select the ONE that applies)

[ ] Solo practice

[ ] Group physician practice

[ ] Interprofessional team-based practice

[ ] Mixed practice (solo and group/or interprofessional practice)

[ ] Other, please specify...: _________________________________________________

8) Which of the following best describes your current practice type?

( ) Comprehensive care (see definition) practicing in one setting only (e.g., community office-based practice only)

( ) Comprehensive care (see definition) practicing in two or more clinical settings (e.g. in-hospital, long-term care, office-based)

( ) Comprehensive care with a special interest (such as chronic pain, care of the elderly, palliative care, etc.) incorporated into practice

( ) Focused practice providing care in one specific clinical area (e.g. sports medicine, emergency medicine)

( ) Other, please specify...: _________________________________________________

9. Do you currently have an academic affiliation?

[ ] Yes

[ ] No

10. Please rate “how well your residency program *prepared* you for the 37 professional activities included in this survey, grouped into 9 domains. *“Prepared” is defined as possessing the competence, confidence, and ability to successfully adapt these competencies in any setting, situation and to any patient population.*

5-Point Likert Scale

1, unprepared; 2, not very well prepared; 3, prepared; 4, well prepared; 5, extremely well prepared

| **Attend to Practice** |
| --- |
| Provide access to care by maintaining a regular schedule with after-hours coverage as part of an overall system of care to the practice |
| Provide virtual care as part of a system of access and continuity for the practice |
| Manage the ‘total care’ of patients providing continuity, follow-up, and coordination |
| Assess and plan for the care needs of the practice in the context of the local community |
| Maintain an electronic medical record for each patient as part of a system of medical documentation for the practice |
| Attend to practice and personal business functions |
| Support and engage with patient safety processes |
| Participate in collaborative and team-based care |
| Manage self-care to support personal well-being and sustainable practice |
| **Comprehensive and inclusive primary care** |
| Provide reproductive care |
| Provide comprehensive continuity-based primary care for children and youth |
| Provide comprehensive continuity-based primary care for adults |
| Provide comprehensive continuity-based primary care for the elderly |
| Provide primary palliative and end-of-life care |
| Manage patients with complex and co-morbid illnesses |
| Provide primary care that addresses the health care needs of diverse peoples as part of a commitment to health equity |
| Provide culturally safe primary care that addresses the specific health care needs of First Nations, Inuit, and Metis people |
| Perform common minor/office procedures (see Core Procedures list) |
| **Maternal & Newborn Care** |
| Provide antepartum care |
| Manage a low-risk labour and delivery |
| Provide postpartum care |
| Provide newborn care in the hospital and community |
| Perform common intrapartum care procedures (see Core Procedures list) |
| **Emergency Care** |
| Assess and manage patients of all ages with common urgent and emergent presentations in all settings |
| Assess and stabilize patients of all ages with life-threatening, high-acuity presentations in all settings |
| Perform commonly required emergency procedures (see Core Procedures list) |
| **Home & Long Term Care** |
| Provide primary care for patients with unique and complex medical needs in the home, long-term care facility and other community-based settings |
| **Hospital Care** |
| Provide medical care in the hospital as the ‘Most Responsible Physician’ |
| Provide surgical assistance in the operating room |
| Perform common in-hospital procedures (see Core Procedures list) |
| **Advocacy** |
| Work with patients to assess and address their social determinants of health |
| Engage with the local community to understand and improve health conditions and access to care |
| **Leadership** |
| Provide leadership in everyday professional practice |
| **Scholarship** |
| Maintain and enhance knowledge to provide care that is evidence-informed and responds to practice needs |
| Participate in QI activities as part of practice improvement |
| Participate in research activities as part of practice improvement |
| Teach and supervise learners in everyday practice functioning as a ‘clinical coach’ (per FTA Framework) |
