# Supplementary material for: Are early career family physicians prepared for practice in Canada? A qualitative study
Source: BMC Med Educ. 2023 May 24;23:370. doi: 10.1186/s12909-023-04250-z (PMC10206365; doi:10.1186/s12909-023-04250-z)
Supplement: Supplementary file 2 — Supplementary Material 2 [file 12909_2023_4250_MOESM2_ESM.docx]

**Supplementary Material 2: Interview Guide**

**Context**

1. Please introduce yourself (without identifying your full name) and describe your practice *(Probe:* location, experience, type of practice, length in practice, residency program).

**Preparedness for Practice**

1. (a) We will be talking about preparedness for practice. How would you define what it means for you to be prepared for independent family medicine practice (Probe: competence, capabilities, adaptability, self-confidence)?
2. Do you feel that you were prepared to enter independent family medicine practice?

(b) If yes, can you share a story within your first few years of practice that demonstrates an example of how you felt prepared to practice independently?

c) How did experiences in your residency program contribute to you feeling prepared? Be specific.

d) If no, can you share a story that describes a time in your first few years of practice when you felt unprepared? What did you do?

e) What aspects related to your residency training contributed to you being prepared/unprepared?

Probe for:

- Curriculum (content, structure)
- Experience
- Supervisors and Mentors (relationships, quality of teaching)
- Culture (setting)

f) What other factors contributed to you feeling prepared/unprepared?

Probe for:

- Organizational factors (resources, opportunities for continuous care, practice support (mentorship, senior support, feedback, continuing professional development, access to extra training, onboarding, shadowing opportunities)
- Personal factors (personality characteristics)

**Family Medicine Professional Profile**

1. (a) The CFPC is promoting the use of the Family Medicine Professional Profile (FMPP) to describe the collective vision of family medicine describing the scope of practice and training for family physicians, and highlights our philosophy of care. As you look at the document (please see attached) to what extent do you think your residency training prepared you to practice family medicine aligned to this vision?

Primary Responsibilities:

- Comprehensive medical care for all people, ages, life stages, and presentations. This care includes all clinical domains, both acute and chronic, and all stages, from preventive to palliative care. Family physicians work across care settings and regulatory environments, including: Primary care, Emergency care, Home and long-term care, Hospital care, Maternal and newborn care
- Leadership
- Scholarship
- Advocacy

Work Arrangements & Settings: PMH models

Special Attributes:

- Relationship- and patient-centred
- Community-adaptive
- Collaborative and continuous

(b) If you felt prepared, what educational factors have enabled your preparedness? If no, what factors served as barriers?

(c) To what extent are you doing the activities outlined in the FMPP? For the activities that you are not doing in practice, please explain why?

(d) For the community-adaptive domain, to what extent do you feel you you have the ability to adapt to changing environments and patient populations? If yes, can you share an example of when you did this in the last few years or if you plan to do this upcoming. What facilitates your ability to be adaptable? What barriers do you face enabling you to adapt to need in the community/or your patients? How might you describe a definition for family physicians who are community adaptive?

1. Do you see yourself in this Family Medicine Professional Profile – why or why not? What would need to happen for you to feel like you as a family physician are contributing to this collective vision of family medicine based upon your own personal approach to the practice of family medicine? How might your residency program better prepare you to work towards this vision? What else would need to happen?

**Practice Intentions versus Practice Choice**

1. (a) When thinking about what you thought your practice of family medicine would look like upon leaving residency, versus now after a few years of practice, how similar or different was your practice intentions to the actual practice you have today? Describe what you thought you would be doing and what you are actually doing?

(b) What factors influenced your practice decisions?

Probe for:

- Personal/lifestyle – likes/dislikes, family decisions, location interest, clinical interests
- Educational – medical school (culture, role models, curriculum, experience), residency (setting, specialized training, Individual competence, confidence)
- Organizational Factors (resources, opportunities for continuous care, Practice Support (mentorship, senior support, continuing professional development, access to extra training, onboarding)
- Policy Environment and Practice Opportunities Factors – models of care, opportunities for specialized practice, population needs, remuneration, license to practice, hospital privileges

**Family Medicine Educational Strategies**

1. How do you see your practice changing over time?
2. What advice would you give to the College to help better prepare family medicine graduates for practice?
3. If you were mentoring a new family medicine resident, what advice would you give them about preparing for a career in family medicine?
4. Anything else that you think is important for us to know?
